# Amygdala and hippocampal contributions to broad autism phenotype: Project Ice Storm

**DOI:** 10.1038/s41398-026-03918-6

**Published:** 2026-03-19

**Authors:** Xinyuan Li, Muhammad Naveed Iqbal Qureshi, David P. Laplante, Guillaume Elgbeili, Sherri Lee Jones, Suzanne King, Pedro Rosa-Neto

**Affiliations:** 1 Integrated Program in Neuroscience, McGill University, Montreal, QC Canada; 2 Douglas Mental Health University Institute, Montreal, QC Canada; 3 Montreal Neurological Institute, Montreal, QC Canada; 4 Department of Neurology and Neurosurgery, McGill University, Montreal, QC Canada; 5 Centre for Child Development and Mental Health, Lady Davis Institute-Jewish General Hospital, Montreal, QC Canada; 6 Department of Psychiatry, McGill University, Montreal, QC Canada

**Keywords:** Neuroscience, Autism spectrum disorders

## Abstract

Prenatal maternal stress (PNMS) increases the risk for autism, and individuals with autism inconsistently exhibit increased or decreased volumes and functional connectivity of the whole amygdala and the whole hippocampus. Given heterogeneous structures of the amygdala and hippocampus and the heterogeneity of autism symptoms, it is worth examining how their subregions contribute to different autism phenotypes. T1-weighted and resting-state functional MRI data were acquired from 32 young adults of mothers who were pregnant during, or within 3 months of, the 1998 Quebec ice storm. Their broad autism phenotype (BAP) was self-reported, including aloof personality, pragmatic language impairment and rigid personality. This sample has a wide range of scores on the BAP Questionnaire. Volumes of the amygdala nuclei and hippocampal subfields were calculated. Seed-to-voxel analysis was applied to examine functional connectivity of the amygdala nuclei and hippocampal subfields with the rest of the brain, and linear regressions were implemented to examine associations of volume and functional connectivity with the three autism phenotypes. Primarily, we found that 1) rigid personality was associated with decreased left hippocampal cornu ammonis (CA)1 volume; 2) pragmatic language impairment was associated with decreased left hippocampal CA1 connectivity with the supplementary motor area, and increased right hippocampal CA4 connectivity with the left putamen; and 3) rigid personality was associated with increased right central amygdala connectivity with the left inferior lateral occipital cortex (LOC); and increased left hippocampal CA3 connectivity with the right superior parietal lobule, increased right hippocampal CA4 connectivity with the left superior LOC, and increased right hippocampal dentate gyrus connectivity with the left superior LOC. In contrast, we found no associations with aloof personality. Our results suggest that, within a sample exposed to PNMS, amygdala and hippocampal structure and function contribute differently to two different autistic-like characteristics, with hippocampus-motor connectivity explaining variance in communication impairment, and with hippocampal volume, amygdala- and hippocampus- sensory connectivity sharing the common mechanism in rigid behaviors. Given these links between brain and autistic-like traits, future research should examine whether brain volumes and connectivity mediate associations between PNMS and autistic-like traits in young adulthood.

## Introduction

Autism is a neurodevelopmental disorder characterized by social and communication deficits and restricted, repetitive behaviors [1]. Individuals with autism vary widely with respect to the defining phenotypes. The phenotypic variability likely reflects diverse etiological mechanisms, and phenotypically diverse individuals may require different treatment strategies [2]. Therefore, it is important to investigate different autistic phenotypes.

Autism can be viewed as the extreme end of the distribution of autistic-like traits in the population [3], and evidence suggests that similar etiological factors apply across the continuum [4, 5]. An extension of the dimensional approach for autism concerns the autism spectrum hypothesis, which proposes a broadening of the spectrum so that not only autistic traits at the clinical level but also those observed in non-clinical populations are included [6]. This extension of the autism trait continuum into the non-clinical population implies the existence of a broad autism phenotype (BAP) that is defined as autistic-like traits that are milder but qualitatively similar to those seen in autism [7]. Compared to the rare prevalence of clinical autism [8], autistic-like traits are more common in the general population [9], making it important to study the BAP which may provide insights into the etiology of autism. The BAP includes three primary phenotypes: aloof personality, pragmatic language impairment and rigid personality. Aloof personality parallels social deficits, and is characterized by a lack of interest in or enjoyment of social interaction. Pragmatic language deficits parallel communication deficits, and are characterized by difficulties in communicating effectively or in holding a fluid, reciprocal conversation. Rigid personality, paralleling restricted, repetitive behaviors, is characterized by little interest in change or difficulty adjusting to change [10].

Despite the complicated etiology, prenatal maternal stress (PNMS) is a potential environmental risk factor of autism [11]. We know for instance that prenatal maternal depression is associated with child autism [12–14], and that prenatal maternal anxiety is associated with non-clinical autistic-like traits in children [15]. Apart from prenatal mood, using natural disasters to examine PNMS has several advantages, primarily because natural disasters are independent events that are not confounded by heritable parental personality traits.

In January 1998, a severe ice storm struck the province of Quebec that led to one of Canada’s worst natural disasters. To evaluate the impact of PNMS on offspring development, we launched Project Ice Storm, the world’s first prospective longitudinal natural disaster PNMS cohort, in June 1998. We recruited women who were pregnant during the crisis or became pregnant within 3 months following the ice storm, and assessed their stress experience including objective hardship, subjective distress and cognitive appraisal. The follow-up assessments of these mother-child dyads began from the age of 6 months, and continued approximately every two years, until 19 years.

Natural disaster-related PNMS research can help determine whether associations between PNMS and autistic-like traits are due more to the objective degree of exposure the pregnant woman endures, her level of subjective distress, or her cognitive appraisal of the event. More severe autistic-like traits in the Project Ice Storm children at age 6½ were associated with greater prenatal maternal objective hardship and subjective distress [16]. At age 19, we found that 21.4% of the variance in BAP total score was explained by maternal objective hardship. In the three BAP phenotypes, 6.8% of the variance in aloof personality was explained by maternal objective hardship; 15.1% of the variance in pragmatic language impairment was explained by maternal subjective distress; 20.0% of the variance in rigid personality was explained by maternal objective hardship and 14.3% by maternal cognitive appraisal [17]. Furthermore, it was shown that DNA methylation of genes related to autism (specifically the PI3K/AKT/mTOR pathway) mediated the effects of maternal objective hardship and cognitive appraisal on the total score as well as on aloof personality and pragmatic language impairment across ages 15, 16, and 19 [18].

Apart from PNMS, neuroimaging findings have partly unveiled neural correlates of autism; the amygdala and hippocampus have been promising regions of interest in autism research [19, 20]. The amygdala plays a central role in emotion processing [21] and social cognition [22]. Cytoarchitectonic mapping of the amygdala in a human post-mortem brain subdivided the amygdala into the basolateral nuclei of the amygdala (BLA) and the centromedial nuclei of the amygdala (CMA) [23]. The BLA are composed of the basal nucleus, lateral nucleus, accessary basal nucleus and paralaminar nucleus [24, 25], and the CMA are composed of the central nucleus of the amygdala (CeA) and medial nucleus of the amygdala (MeA) [23, 25]. The basal nucleus and lateral nucleus receive multi-sensory inputs from widespread cortical and subcortical regions, and send major projections to the medial temporal lobe and prefrontal cortex. The central nucleus and medial nucleus send primary projections to the hypothalamus, bed nucleus of the stria terminalis and several nuclei in the midbrain. In agreement with their afferent and efferent connections, the BLA are mainly involved in social cognition and emotion generation [26], and the CMA regulate autonomic, hormonal and behavioral responses [27].

Hippocampal contribution to social cognition [28, 29] makes it another candidate in the context of autism. It has been shown that social impairment, such as poor social skills and social withdrawal, often co-occur with hippocampal structural and functional abnormalities [30]. One observed hippocampal involvement is social cognitive mapping that makes predictions and supports adaptive decision-making. Specifically, maps of social relationships help one predict the actions of others and respond appropriately, just as maps of physical locations help one navigate flexibly within a changing environment [31]. Meanwhile, the traditional hippocampal role in memory and learning [32] cannot be neglected. While memory dysfunction is not the most prominent characteristic of autism, atypical memory and learning in autistic individuals have been reported; autistic individuals, for instance, have difficulties recalling episodic memories [33], have diminished false memory and reduced integration of source memory and thinking [34] and have difficulties in reversal learning [35]. Further, it has been reported that rigid memory, inflexible retrieval and less relational learning may induce atypical behaviors (e.g., restricted, repetitive behaviors) [36]. Similar to the amygdala, the hippocampus is a heterogeneous structure divided into multiple subfields with distinct functional roles. The hippocampal cornu ammonis (CA)3, CA4 and dentate gyrus (DG) are involved in learning and memory encoding while hippocampal CA1 is involved in memory retrieval [37].

Numerous structural MRI studies have inconsistently shown atypical volumes of the amygdala and hippocampus in autistic individuals compared to typically-developing controls, including larger volumes of the whole amygdala [38–40] and whole hippocampus [38, 41–43], or smaller volumes of the whole amygdala [44, 45] and whole hippocampus [44], or no volumetric differences in the whole amygdala [41, 43] and whole hippocampus [46–48]. The discrepancy might be attributed to the age at which the assessments occurred. Subsequent studies [43, 49] demonstrate that autistic children had larger left and right whole amygdala volumes than typically-developing children while there were no differences in left or right whole amygdala volume in late adolescence and young adulthood. Further, there are conflicting findings on associations between the three features of autism (i.e., social and communication deficits, repetitive behaviors) and whole amygdala and whole hippocampal volumes [38, 40–42]. Considering heterogeneous structures of the amygdala and hippocampus, further investigation into their subregions may partly explain these inconclusive findings. One recent study [50], for example, reported that increased BLA volume was associated with social deficits, and that increased CeA volume was associated with restricted, repetitive behaviors. In terms of hippocampal subfields, abnormal CA1, CA3, CA4 and DG volumes have been seen in individuals with autism [51], while their associations with the three phenotypes of autism have yet to be examined.

In comparison, less is known about how varying degrees of autistic-like traits correspond to brain structural alterations in non-clinical samples. One study [52] reported prediction of subclinical autistic traits by large whole amygdala volume and small whole hippocampus volume during adolescence. Another study [53] showed positive correlations between autistic-like traits and the volume of whole amygdala-superior temporal sulcus white matter connectivity in young adults. In addition, we previously studied 19-year-old young adult offspring from Project Ice Storm and typically-developing non-exposed controls without autism, and we found that Project Ice Storm participants had larger volumes of the basal nucleus, lateral nucleus, central nucleus and medial nucleus of the amygdala as well as larger volumes of the hippocampal CA1, CA3, CA4 and DG [54]. These findings indicate that neural correlates of autistic traits also seem to lie on a continuum in the non-clinical population. However, there is scant knowledge about how structural changes in amygdala and hippocampal subfields contribute differently to explain variance in autistic-like traits, specific to different phenotypes. Given the fact that autism and autistic-like traits are etiologically linked, one study [50] showing positive associations between BLA volume and social deficits, and between CeA volume and repetitive behaviors, provides hypotheses for these two domains of subthreshold autistic characteristics.

In addition, there is emerging evidence from resting-state functional MRI research that individuals with autism, compared to typically-developing controls, showed lower BLA functional connectivity with the superior parietal lobe [25], the nucleus accumbens [55] and the ventromedial prefrontal cortex [24], and higher CMA functional connectivity with the occipital cortex [55]. Compared to a growing body of research on functional connectivity alterations of amygdala nuclei in individuals with autism, our knowledge about functional connectivity of hippocampal subfields is limited. Further, it remains unclear the extent to which amygdala and hippocampal functional connectivity explain variance in autism phenotypes.

To address these gaps, the present study aimed to determine: 1) associations between amygdala and hippocampal subregion volumes and BAP; and 2) associations between amygdala and hippocampal subregion functional connectivity and BAP. We hypothesized that 1) increased BLA and CMA volumes would be associated with higher BAP total score; increased CA1, CA3, CA4 and DG volumes would be associated with higher BAP total score; regarding the three subdomains of BAP, increased BLA volume would be associated with aloof personality, and increased CMA volume would be associated with rigid personality; and 2) atypical functional connectivity of the BLA and CMA would be associated with higher BAP total score; atypical functional connectivity of the CA1, CA3, CA4 and DG would be associated with higher BAP total score. Regarding the three subdomains of BAP, atypical BLA functional connectivity would be associated with aloof personality, and atypical CMA functional connectivity would be associated with rigid personality. In contrast to the specific hypotheses of the BLA and CMA on the three subdomains of BAP, we were unable to propose prior specific hypotheses on the CA1, CA3, CA4 and DG.

## Methods

### Participants

This study is part of Project Ice Storm (described in Introduction). We recruited women who were pregnant during the ice storm or became pregnant within 3 months after, and assessed their stress experience including objective hardship, subjective distress and cognitive appraisal. These PNMS measures are described in detail elsewhere [17]. At age 19, 33 young adults self-reported their BAP score. One participant was excluded because of missing MRI data. Thirty-two participants (21 females) were included in all analyses (mean (SD) age = 18.75 (0.36) years; range 18.10–19.62 years). Table 1 presents the demographic and descriptive information. Given previous Project Ice Storm findings on autistic-like traits across different developmental ages (i.e., ages 6 [16], 15, 16 and 19 [17, 18]), and given the wide range of BAP trait scores reported in Table 1, this sample is ideal for testing our hypotheses.

**Table 1:** Demographic characteristics of 32 young adult participants.

	Range / % (n)	Mean ± SD
Parental SES		
Middle class	28.1% (9)	
Upper middle class	46.9% (15)	
Upper class	25% (8)	
Sex (F)	65.6% (21)	
Gestational age at birth (week)	33.43 – 41	39.51 ± 1.36
Birth weight (g)	1850 – 4432	3506.16 ± 491.66
Age at scan (year)	18.10 – 19.62	18.75 ± 0.36
IQ ^a^	90 – 137	113.67 ± 11.14
Handedness (R)	90.6% (29)	
Broad autism phenotype ^b^		
Total score	1.53 – 4.44; 34.4% (11)	2.80 ± 0.70
Aloof personality	1.33 – 4.73; 34.4% (11)	2.95 ± 0.99
Rigid personality	1.83 – 4.83; 37.5% (12)	3.15 ± 0.78
Pragmatic language impairment	1 - 4.33; 28.1% (9)	2.30 ± 0.70

^a^IQ was assessed at 19 years of age using the Wechsler Adult Intelligence Scale – Third Edition short form.

^b^% (n) represents the percentage of the participants meeting the clinical cutoffs for broad autism phenotype.

*F* female, *IQ* intelligence quotient, *L* left, *M* male, *R* right, *SD* standard deviation, *SES* socioeconomic status.

### Ethical approval

This study was approved by the Douglas Mental Health University Institute Research Ethics Board. We obtained written informed consent from all participants at all phases of the study. All methods were performed in accordance with the relevant guidelines and regulations.

### Broad autism phenotype

The Broad Autism Phenotype Questionnaire (BAPQ) [56] was designed to assess BAP in non-clinical populations, and has been validated against direct clinical assessment of BAP [7]. The self-report BAPQ includes 36 questions assessing three subscales of 12 questions each: Aloof Personality, Pragmatic Language and Rigid Personality. Aloof personality is defined as a lack of interest in, or enjoyment of, social interaction (corresponding to social deficits of autism). Pragmatic language impairment is defined as deficits in social aspects of language, resulting in difficulties communicating effectively or in holding a fluid, reciprocal conversation (corresponding to communication deficits of autism). Rigid personality is defined as little interest in change or difficulty adjusting to change (corresponding to restricted, repetitive behaviors of autism) [10]. Questions are rated using a 6-point Likert scale ranging from “very rarely” (1) to “very often” (6) [7], and mean ratings are calculated for each score. Higher scores represent more severe traits. The following scores are used as clinical cutoffs for the total score (3.15), aloof personality (3.25), pragmatic language impairment (2.75) and rigid personality (3.50) [10]. Inter-item reliability for each subscale was examined: Cronbach’s α coefficient was 0.94 for the aloof subscale, 0.91 for the rigid subscale, 0.85 for the pragmatic language subscale and 0.95 across all 36 items [10]. Inter-item reliability did not differ between self- and informant-report versions. Sensitivity and specificity were at or above 70% for all subscales and over 80% for aloof personality and rigid personality. Sensitivity and specificity were approximately 80% for the total BAPQ score [10].

### MRI data acquisition

MRI data were acquired using a 3.0 T Siemens MAGNETOM Trio TIM Syngo MRI scanner (Siemens, Erlangen, Germany), with a 12-channel head coil. Anatomical images were obtained using a 3D, T1-weighted (T1w) Magnetization Prepared Rapid Gradient Echo sequence (192 slices, TR = 2400 s, TE = 2.43 ms, slice thickness = 1 mm, Flip Angle = 8°, matrix = 256 × 256). Resting-state functional images were acquired using a T2*-weighted echo-planar imaging sequence (42 slices, TR = 2600 ms, TE = 30 ms, Flip Angle = 90°, slice thickness = 3.4 mm, FoV = 218 mm, matrix = 64 × 64). Throughout the 5:01-minute resting-state scan, participants were instructed to lie still with their eyes open.

### MRI data preprocessing

fMRIPrep v.1.5.7 [57] was used for preprocessing. The T1w image was corrected for intensity non-uniformity [58], and used as T1w-reference throughout the workflow. The T1w-reference was then skull-stripped. Brain tissue segmentation of gray matter, white matter (WM) and cerebrospinal fluid (CSF) was performed on the brain-extracted T1w [59]. Volume-based spatial normalization to the montreal neurological institute (MNI) space was performed through nonlinear registration, using brain-extracted versions of both T1w reference and the T1w template. For each of the blood-oxygen-level-dependent (BOLD) runs found per subject, first, a reference volume and its skull-stripped version were generated. A deformation field to correct for susceptibility distortions was estimated based on fMRIPrep’s fieldmap-less approach. Based on the estimated susceptibility distortion, a corrected echo-planar imaging reference was calculated for a more accurate co-registration with the anatomical reference. The BOLD reference was then co-registered to the T1w reference [60]. Co-registration was configured with nine degrees of freedom to account for distortions remaining in the BOLD reference. The BOLD time-series were resampled onto their original, native space by applying a single, composite transform to correct for head-motion and susceptibility distortions. The BOLD time-series were then resampled into the MNI space. Automatic removal of motion artifacts using independent component analysis (ICA-AROMA) [61], was performed on the spatially-normalized, preprocessed BOLD on MNI space time-series after removal of non-steady state volumes and spatial smoothing with an isotropic, Gaussian kernel of 6 mm FWHM. Finally, we conducted WM and CSF signal removal from the BOLD time series and temporally bandpass filtering (>0.01 Hz).

### FreeSurfer segmentation

**Fig. 1:**
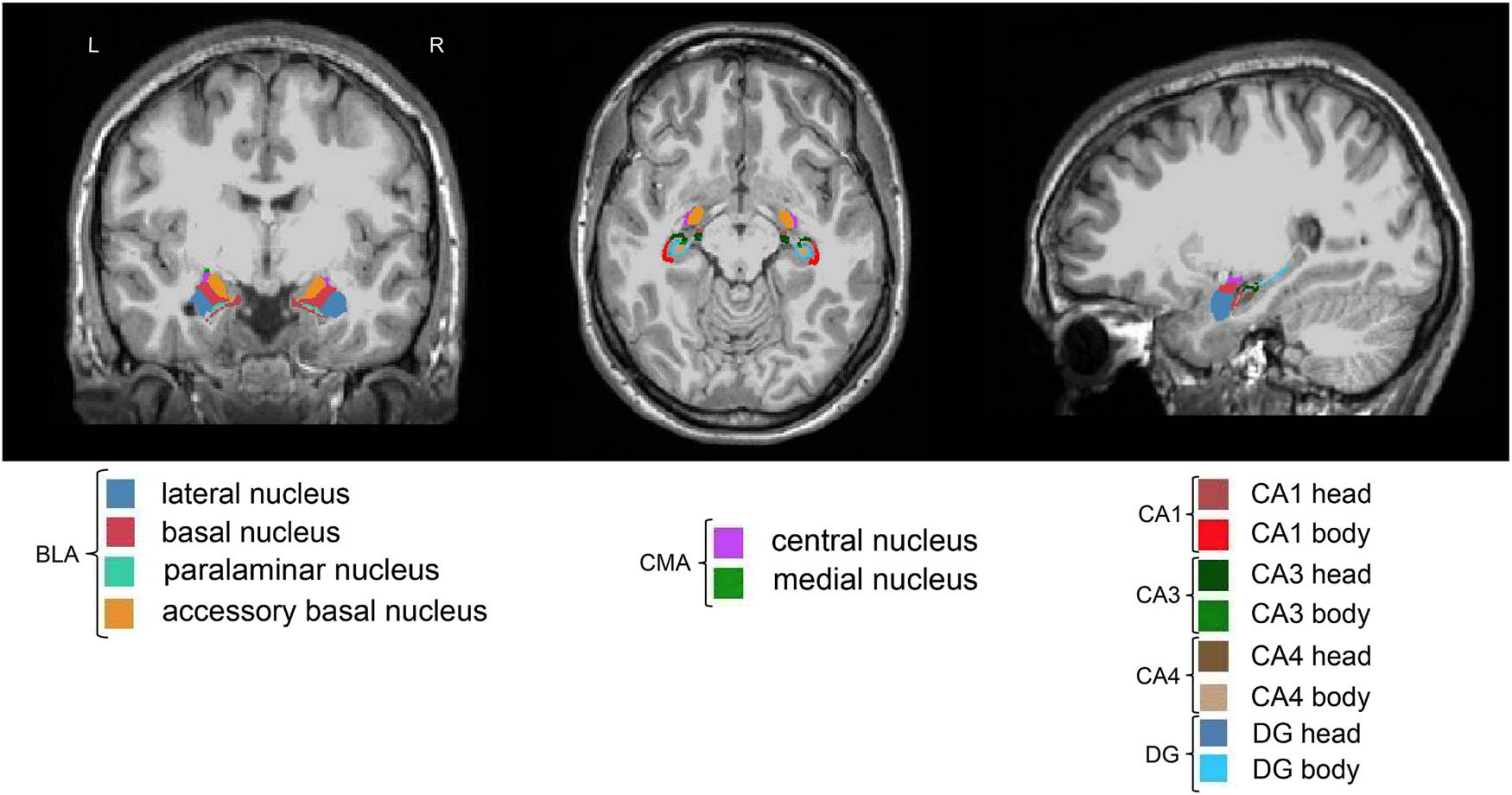
Segmentation of the hippocampus and the amygdala of one participant. The column from left to right represents coronal, axial and sagittal views, respectively. The amygdala nuclei (BLA and CMA) and hippocampal subfields (CA1, CA3, CA4 and DG) (left and right) were labeled with different colors. BLA, basolateral nuclei of the amygdala; CA, cornu ammonis; CMA, centromedial nuclei of the amygdala; DG, dentate gyrus; L, left; R, right.

fMRIPrep preprocessed T1-weighted brain regions in MNI space were segmented using FreeSurfer v.7.1.1 [62] and its library tool *recon-all*. The segmentation of the amygdala and the hippocampus was performed using *segmentHA_T1.sh* [63, 64]. The segmentation of 9 amygdala nuclei and 19 hippocampal subfields is presented in our previous research [54]. Given BLA and CMA have been commonly investigated in neuroimaging research of autism, AFNI *3dcalc* was used to combine the BLA and CMA: the BLA are composed of lateral nucleus, basal nucleus, accessory basal nucleus and paralaminar nucleus and the CMA are composed of central nucleus and medial nucleus [23–25, 65]. Among the 19 hippocampal subfields, only CA1, CA3, CA4 and DG were included in the current study given their common exploration in neuropathology of autism. AFNI *3dcalc* was also used to combine the head and body of hippocampal CA1, CA3, CA4 and DG subfields. The BLA, CMA, CA1, CA3, CA4 and DG are shown in Fig. 1. The quality of FreeSurfer segmentation was visually inspected using FreeView by two authors (X.L. and M.N.I.Q).

### Volume analysis

Volumes of the amygdala nuclei (i.e., BLA and CMA) and the hippocampus subfields (i.e., CA1, CA3, CA4 and DG) for each participant were computed with FreeSurfer v.7.1.1. All subsequent analyses were conducted using IBM SPSS v.26. Linear regression analyses were conducted to examine associations between the volumes and BAP scores (i.e., total score and three subdomain scores). Given the presence of autism or autistic-like traits in a sex-specific manner, sex was controlled for as the covariate in the volume analyses. To examine the association between the volumes and BAP scores independent of PNMS, we implemented a residualization approach. First, we regressed BAP scores on three PNMS measures (i.e., objective hardship, subjective distress and cognitive appraisal) and saved the residuals. Second, we regressed the volumes on the same PNMS measures and saved the residuals. Finally, these residuals were then entered into linear regression analyses, with residualized volumes as the dependent variable, residualized BAP scores as the independent variable, and sex as the covariate.

### Functional connectivity analysis

Seed-to-voxel analyses were conducted using the CONN toolbox v.19b [66]. The BLA, CMA, hippocampal CA1, CA3, CA4 and DG (left and right, separately) computed with FreeSurfer v.7.1.1 were used as seeds for the amygdala nuclei and hippocampal subfields for each participant. Pearson correlation coefficients were calculated between average BOLD time series extracted from the above subregion seeds (left and right, separately) and the time courses of all voxels across the brain. The resultant correlation coefficients were converted to normally distributed mean z-values. At the group level, linear regression analyses were conducted with mean z-values as the dependent variable and with BAP scores (i.e., total and three subdomain scores) as the independent variable, and with sex as the covariate. Thresholds for the group-level results were set to *p* < 0.001 uncorrected for voxel, FDR-corrected *p* < 0.05 for cluster.

To examine the association between amygdala and hippocampal functional connectivity and BAP scores independent of PNMS, we implemented a residualization approach. First, we regressed BAP scores on three PNMS measures and saved the residuals. Second, we regressed the functional connectivity on the same PNMS measures and save the residuals. Finally, these residuals were then entered into linear regression analyses, with residualized functional connectivity as the dependent variable, residualized BAP scores as the independent variable, and sex as the covariate.

## Results

### Associations between amygdala and hippocampal volumes and BAP total score

No associations were observed between BAP total score and either BLA volume or CMA volume, nor with hippocampal subfield volumes.

### Associations between amygdala and hippocampal volumes and BAP subdomain scores

**Fig. 2:**
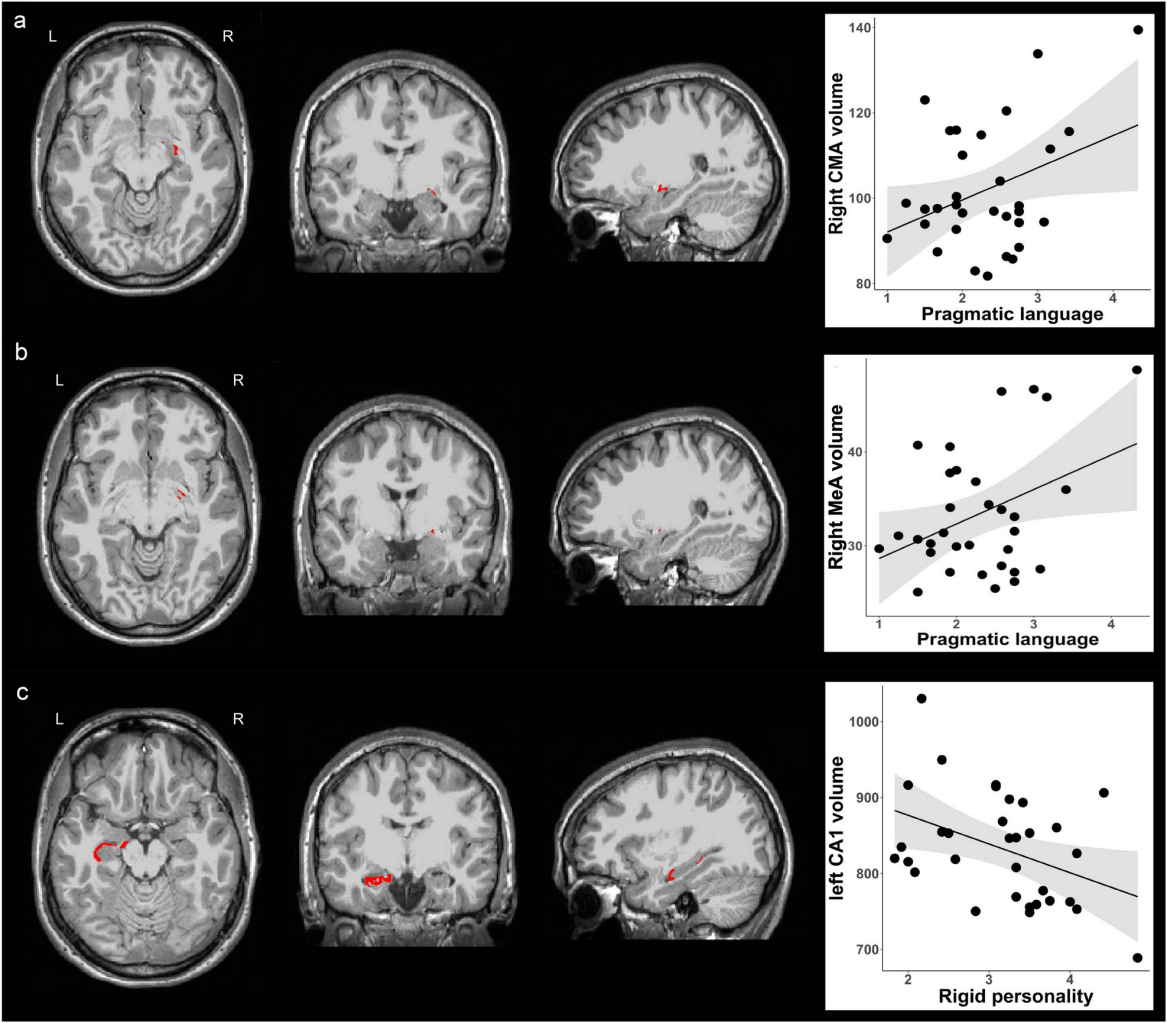
Correlations of amygdala and hippocampal subregion volumes with two broad autism phenotypes. (**a**) The left three columns from left to right represents axial, coronal and sagittal views of the right CMA. The right column of the scatterplot represents positive association between the right CMA volume and pragmatic language impairment. (**b**) The left three columns from left to right represents axial, coronal and sagittal views of the right MeA. The right column of the scatterplot represents positive association between the right MeA volume and pragmatic language impairment. (**c**) The left three columns from left to right represents axial, coronal and sagittal views of the left CA1. The right column of the scatterplot represents negative association between the left CA1 volume and rigid personality. CA, cornu ammonis; CMA, centromedial nuclei of the amygdala; MeA, medial nucleus of the amygdala; L, left; R, right.

No associations were observed for BLA volume and BAP subdomain scores. We found a positive association for CMA volume: increased right CMA volume was associated with pragmatic language deficits (Beta = 0.365, B = 7.533, se = 3.536, *p* = 0.042, Fig. 2a). This association became non-significant after performing residualization (Table S1). When we further explored subnuclei of the right CMA, we found that increased right CMA volume was driven by the right MeA volume, but not the right CeA volume, such that increased right MeA volume was associated with pragmatic language impairment (Beta = 0.385, B = 3.676, se = 1.631, *p* = 0.032, Fig. 2b). After residualization for PNMS, the association between the right MeA volume and pragmatic language remained marginally significant (Table S1 and Fig. S2b). In addition, we found that decreased left hippocampal CA1 volume was associated with rigid personality (Beta = −0.406, B = −37.843, se = 15.755, *p* = 0.023, Fig. 2c), but no associations were observed for other hippocampal subfield volumes and BAP subdomain scores. This association was still significant following residualization (Table S1 and Fig. S2c).

### Associations between amygdala and hippocampal functional connectivity and BAP total score

No associations were observed between BAP total score and BLA functional connectivity, nor with CMA functional connectivity, nor with hippocampal subfield functional connectivity.

### Associations between amygdala and hippocampal functional connectivity and BAP subdomain scores

**Fig. 3:**
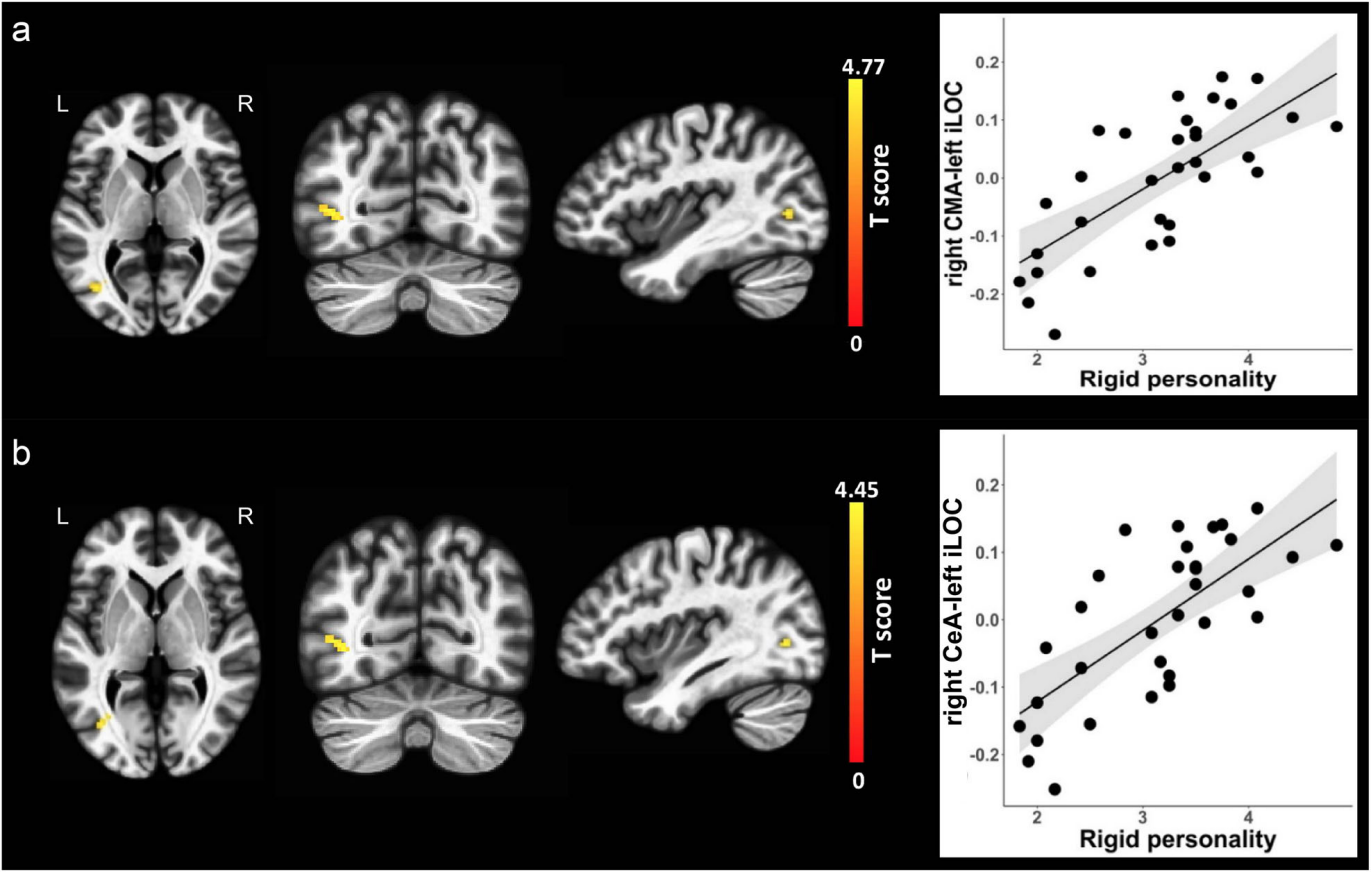
Correlations of amygdala subregion functional connectivity with rigid personality. (**a**) The left three columns from left to right represents axial, coronal and sagittal views of the left inferior lateral occipital cortex. The right column of the scatter plot represents positive association between right CMA functional connectivity with the left inferior lateral occipital cortex and rigid personality. (**b**) The left three columns from left to right represents axial, coronal and sagittal views of the left inferior lateral occipital cortex. The right column of the scatter plot represents positive association between right CeA functional connectivity with the left inferior lateral occipital cortex and rigid personality. Clusters displaying positive associations are depicted in yellow colors. CMA, centromedial nuclei of the amygdala; CeA, central nucleus of the amygdala; iLOC, inferior lateral occipital cortex, L, left; R, right.

No associations were observed for BLA functional connectivity and BAP subdomain scores. No associations were found for CMA functional connectivity and scores of the two BAP subdomains (i.e., aloof personality and pragmatic language impairment). However, we found that increased functional connectivity between the right CMA and one cluster with its peak voxel located at the left inferior lateral occipital cortex (T score = 4.77, p-FDR = 0.006) was associated with rigid personality (Beta = 0.708, B = 0.109, se = 0.019, Fig. 3a). When we further explored subnuclei of the right CMA, we found that increased functional connectivity between the right CeA and one cluster with its peak voxel located at the left inferior lateral occipital cortex (T score = 4.45, p-FDR = 0.015) was associated with rigid personality (Beta = 0.705, B = 0.106, se = 0.019, Fig. 3b). Both associations remained significant after residualization (Table S1 and Fig. S3).

**Fig. 4:**
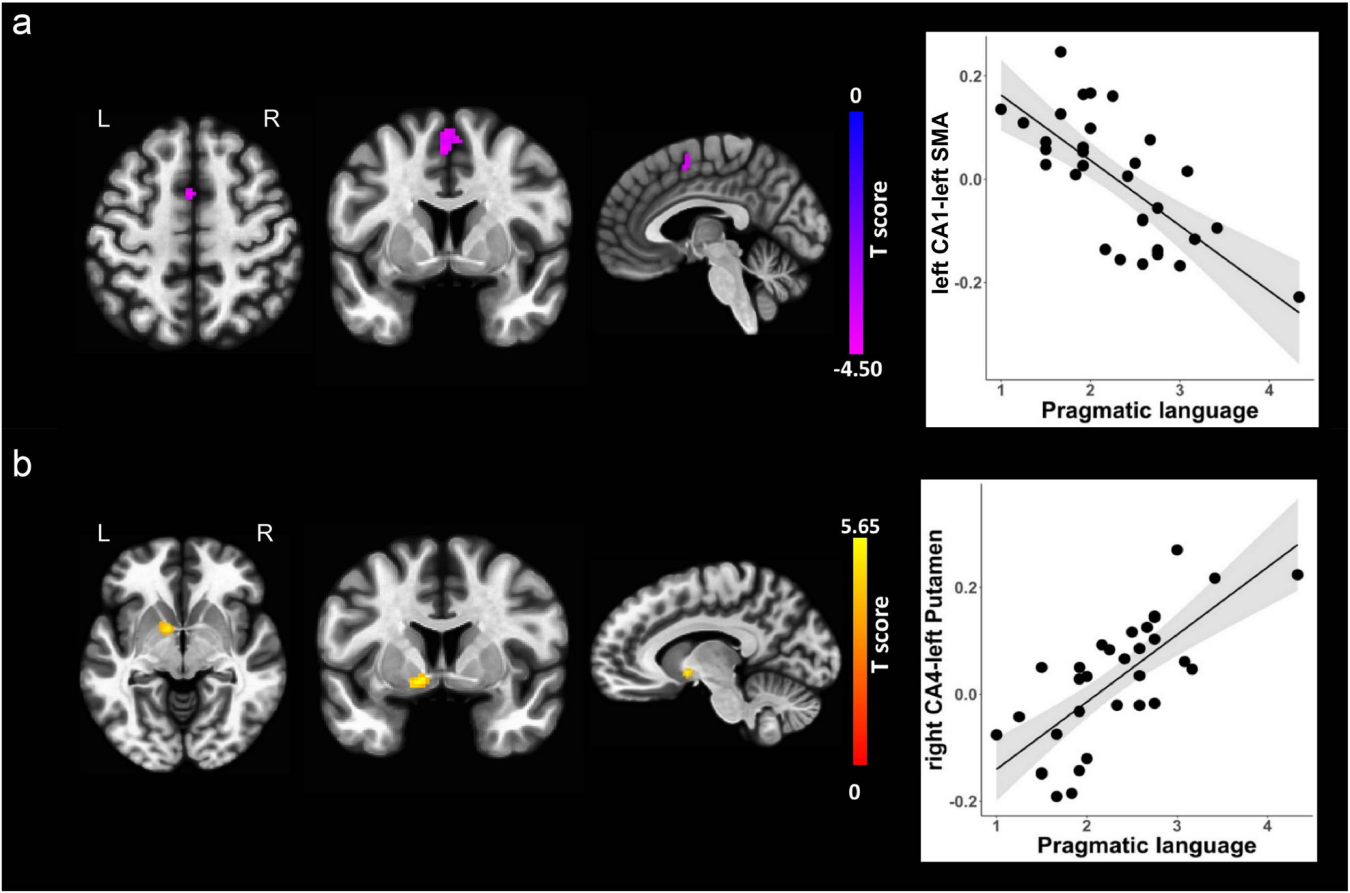
Correlations of hippocampal subregion functional connectivity with pragmatic language impairment. (**a**) The left three columns from left to right represents axial, coronal and sagittal views of the left supplementary motor area. The right column of the scatter plot represents negative association between left CA1 functional connectivity with the left SMA and pragmatic language impairment. (**b**) The left three columns from left to right represents axial, coronal and sagittal views of the left putamen. The right column of the scatter plot represents positive association between right CA4 functional connectivity with the left putamen and pragmatic language impairment. Clusters displaying positive associations are depicted in yellow colors, and clusters displaying negative associations are depicted in purple colors. CA, cornu ammonis; L, left; R, right; SMA, supplementary motor area.

**Fig. 5:**
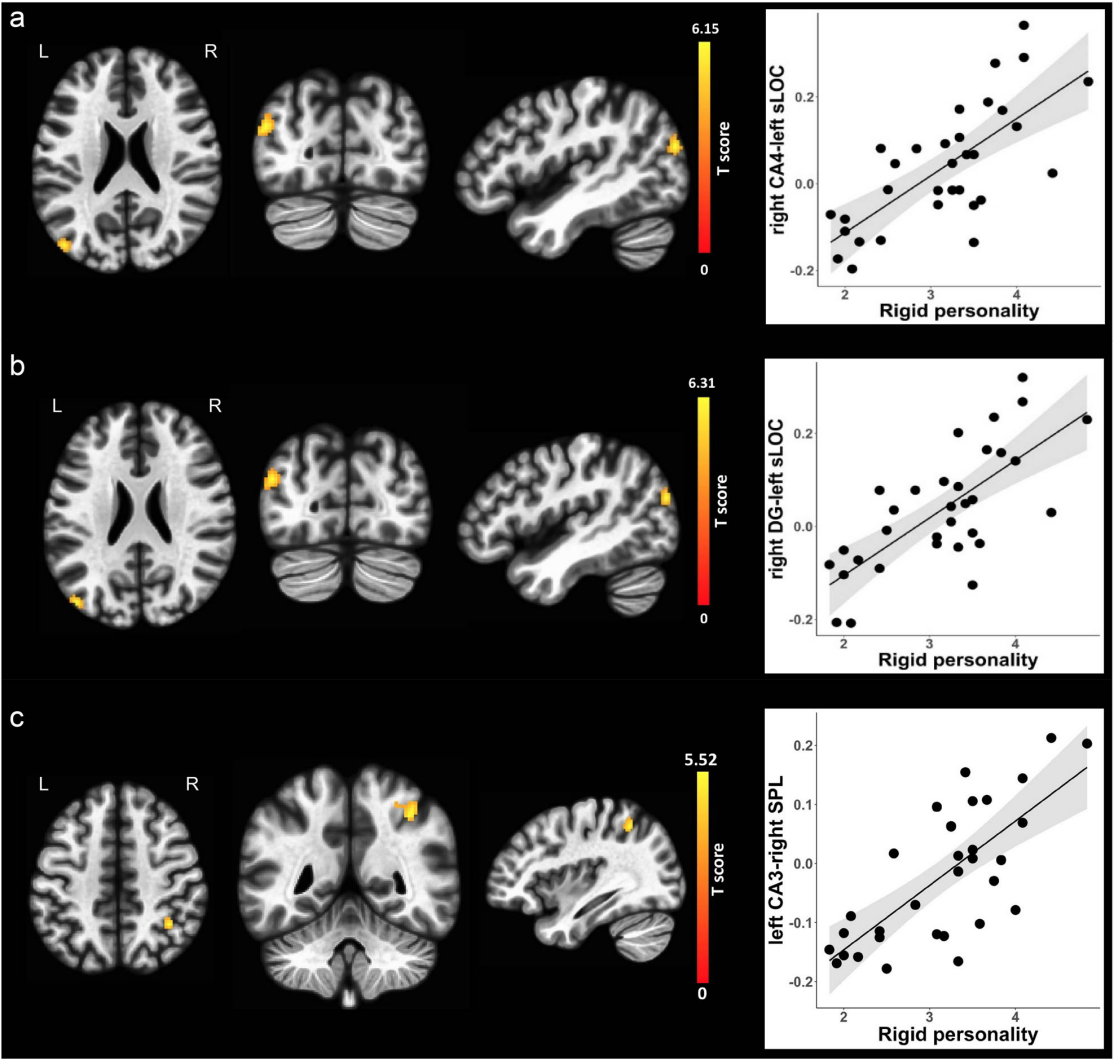
Correlations of hippocampal subregion functional connectivity with rigid personality. (**a**) The left three columns from left to right represents axial, coronal and sagittal views of the left superior lateral occipital cortex. The right column of the scatter plot represents positive association between right CA4 functional connectivity with left superior lateral occipital cortex and rigid personality. (**b**) The left three columns from left to right represents axial, coronal and sagittal views of the left superior lateral occipital cortex. The right column of the scatter plot represents positive association between right DG functional connectivity with the left superior lateral occipital cortex and rigid personality. (**c**) The left three columns from left to right represents axial, coronal and sagittal views of the right superior parietal lobule. The right column of the scatter plot represents positive association between left CA3 functional connectivity with the right superior parietal lobule and rigid personality. Clusters displaying positive associations are depicted in yellow colors. CA, cornu ammonis; DG, dentate gyrus; L, left; R, right; sLOC, superior lateral occipital cortex; SPL, superior parietal lobule.

No associations were observed for hippocampal subfield functional connectivity and aloof personality scores, although we found associations between hippocampal subfield functional connectivity and scores of the two BAP subdomains (i.e., pragmatic language impairment and rigid personality). Specifically, decreased functional connectivity between the left hippocampal CA1 and one cluster involving left and right supplementary motor area (SMA) with its peak voxel located at the left SMA (T score = −4.50, p-FDR = 0.012) was associated with pragmatic language impairment (Beta = −0.715, B = −0.126, se = 0.023, Fig. 4a), while increased functional connectivity between the right hippocampal CA4 and one cluster with its peak voxel located at the left putamen (T score = 5.65, p-FDR = 0.039) was associated with pragmatic language impairment (Beta = 0.735, B = 0.126, se = 0.020, Fig. 4b). In addition, increased functional connectivity between the right hippocampal CA4 and one cluster with its peak voxel located at the left superior lateral occipital cortex (T score = 6.15, p-FDR = 0.007) was associated with rigid personality (Beta = 0.725, B = 0.131, se = 0.024, Fig. 5a); increased functional connectivity between the right hippocampal DG and one cluster with its peak voxel located at the left superior lateral occipital cortex (T score = 6.31, p-FDR = 0.006) was associated with rigid personality (Beta = 0.734, B = 0.124, se = 0.022, Fig. 5b); and increased functional connectivity between the left hippocampal CA3 and one cluster with its peak voxel located at the right superior parietal lobule (T score = 5.52, p-FDR = 0.013) was associated with rigid personality (Beta = 0.724, B = 0.109, se = 0.019, Fig. 5c). The details for the functional connectivity clusters are presented in Table 2. All reported hippocampal subfield functional connectivity associations remained significant after residualization for PNMS (Table S1 and Fig. S4 and Fig. S5).

**Table 2:** Group-level linear results for functional connectivity clusters associated with the two broad autism phenotypes (i.e., pragmatic language impairment and rigid personality) (voxel-level uncorrected *p* < 0.001, cluster FDR-corrected *p* < 0.05).

Clusters	Cluster size	Peak voxel	Additional regions	MNI coordinates	T score ^a^	p-uncorrected	p-FDR
Seed: Right CMA							
	91	Left inferior lateral occipital cortex		−50, −76, 8	4.77	0.0001	0.006
Seed: Right central nucleus							
	73	Left inferior lateral occipital cortex		−38, −70, 2	4.45	0.0005	0.015
Seed: Left CA1							
	87	Left supplementary motor area		−4, 4, 54	−4.50	0.0004	0.012
			Right supplementary motor area				
Seed: Left CA3							
	87	Right superior parietal lobule		36, −50, 48	5.52	0.0003	0.013
Seed: Right CA4							
	90	Left superior lateral occipital cortex		−46, −80, 22	6.15	0.0002	0.007
	62	Left Putamen		−10, 4, −6	5.65	0.0013	0.039
Seed: Right DG							
	92	Left superior lateral occipital cortex		−46, −80, 24	6.31	0.0002	0.006

^a^Positive T scores denote positive associations with broad autism phenotype; negative T scores denote negative associations with broad autism phenotype.

*CA* cornu ammonis, *CMA* centromedial nuclei of the amygdala, *DG* dentate gyrus, *FDR* false discovery rate, *MNI* Montreal Neurological Institute.

## Discussion

This study examined associations between the severity of BAP traits and the volumes and functional connectivity of amygdala nuclei and hippocampal subfields in young adults. All participants in this sample were born following a natural disaster and, while a nonclinical sample, there was considerable variance in the severity of BAP traits which allowed us to uncover correlational associations with amygdala and hippocampal structure and function. Our results demonstrate that it is critical to study the different subdomains of BAP traits separately since we found different patterns in their associations with amygdala and hippocampal volumes and functional connectivity, discussed as below.

First, we found no correlations between the overall BAP severity and the volumes and functional connectivity of the amygdala and hippocampal subregions. The lack of association with BLA volume and connectivity echoes previous studies reporting no link between the severity of autism total score and BLA functional connectivity [25], whereas the severity of one specific subdomain of autism symptoms (e.g., social deficits) was associated with BLA volume and functional connectivity in autistic individuals [50, 55]. Similarly, no associations were observed between the overall BAP severity and volumes and functional connectivity of the CMA, hippocampal CA and DG. Together, these findings emphasize the necessity to examine the extent to which structure and function of the amygdala and hippocampal subregions are associated with the severity of specific subdomains of autistic-like traits, instead of the overall severity of autism traits.

Regarding the subdomains of BAP traits, no associations were found in our study between the severity of aloof personality and volumes or functional connectivity of the amygdala and hippocampal subregions. In contrast to our hypotheses, no associations were observed between the severity of aloof personality and BLA volume and functional connectivity, which appears inconsistent with the involvement of the BLA in social cognition [26]. A previous study [50] showed that, in 23 adolescents with autism spectrum disorder, increased BLA volume was associated with high reciprocal social interaction scores on the Autism Diagnostic Interview-Revised. The inconsistency between this previous study [50] and our study might be explained by differences in the samples (nonclinical participants with a range of autistic-like traits versus clinically-diagnosed autistic individuals). Nevertheless, both studies have the limitation of small sample size, and thus, future studies with larger sample are warranted to verify the role of the BLA in social cognition. Another possible explanation is that the BLA is heterogeneous complex composed of four different subnuclei (i.e., basal nucleus, lateral nucleus, accessary basal nucleus and paralaminar nucleus) [24, 25], and that the basal nucleus and lateral nucleus might have closer involvement in social information processing [26]. Therefore, there is also a need to further explore correlations of the two subnuclei structure and function with social deficits. Less surprisingly, we found that the severity of aloof personality was not associated with the CMA volume and functional connectivity possibly due to its closer association with the regulation of autonomic, hormonal and behavioral responses [27], rather than social information processing, despite reciprocal interconnections between the CMA and BLA [26]. As with the amygdala subregions, we found no associations between the severity of aloof personality and the hippocampal CA and DG volumes and functional connectivity, which might be explained by its primary function of memory [32].

Although we found no associations between the severity of aloof personality and structure and function of the amygdala and hippocampal subregions, there were several associations observed between the severity of the two subdomains of BAP traits (i.e., pragmatic language impairment and rigid personality) and structure and function of the amygdala and hippocampal subregions.

Regarding deficits in pragmatic language, the residualization analysis suggests that the association between the right CMA and pragmatic language impairment may be influenced by PNMS. Nonetheless, increased right MeA volume remained marginally significantly associated with pragmatic language impairment after residualization. Pragmatic language impairment primarily refers to deficits in communication, for example, individuals may disrupt a conversation, provide excessive detail during conversations, frequently lose track of the conversation and engage in conversational tangents [67]. The MeA is a primary center for social communication processing; the MeA receives chemosensory input from the main olfactory bulbs projecting to the basal forebrain to elicit appropriate behavioral and physiological responses [68]. Notably, although conventional wisdom suggests that increased volume could afford better processing, we found that larger amygdala volume was associated with more severe pragmatic language impairment. This paradoxical finding has relevance for two competing theories about brain development trajectories in autism; “Early Brain Overgrowth” proposes accelerated brain growth in autistic toddlers followed by abnormally showed growth in older autistic children and adolescents [69], while “Over-Pruning” theory suggests that overly excessive synaptic pruning is predictive of autistic traits, particularly sensory and motor abnormalities [70]. A recent study [71] using two large-sample datasets shows that substantially enlarged brain volume in autistic individuals, compared to typically-developing controls, persists from childhood through young adulthood. Our finding aligns more with the overgrowth theory and somewhat challenge the over-pruning hypothesis. Further exploration is needed to determine how these theories might apply to the non-clinical autistic-like traits described in the current study.

We also found that decreased connectivity of the left hippocampal CA1 with the left SMA and increased connectivity of the right hippocampal CA4 with the left putamen were associated with pragmatic language deficits, possibly reflecting memory-motor-language interface. Although hippocampal implication in pragmatic language impairment may appear unexpected given its classic function in memory, a growing literature reveals that the hippocampus is potentially a key contributor to the processing of language and verbal communication [72–74]. One view proposes the interface between memory and language, such that the hippocampal computations that are critical in memory function also meet many of the demands of flexible language use and processing [73, 75, 76]. For example, the theta-power in the human hippocampal complex involved in memory increases with the establishment of a meaningful context in a sentence [76], and patients with hippocampal amnesia exhibit deficits in the flexible and on-line use of language [77, 78]. In support of this view, our finding of the involvement of the CA1 and CA4 subfields in language inflexibility can be explained by human studies showing that the CA1 and CA4 have sensitive roles in the process of verbal memory retrieval [79]. The hippocampus may support the ability to predict upcoming words by drawing on long-term memory representations of individual words, patterns of lexical co-occurrence and syntactic probabilities [75]. Another proposal is that the hippocampal contribution to social cognition [28] may also underlie effective communication: the hippocampus has been found to track social information in the physical environment, make predictions about the action of others and support appropriate responses and decision-making [30, 80]. Compared to this relatively novel role for the hippocampus, frequently-reported brain regions closely related to social cognition (referred to as “social brain”) involve other regions such as the medial orbitofrontal cortex and the right insula [81]. Given social impairment as the core symptomatology domain of autism, future investigation is needed to gain a comprehensive picture of the neural circuits underpinning social processing.

Meanwhile, our results showing that the motor-related regions (i.e., the left SMA and the left putamen) are implicated in pragmatic language deficits are supported by their previously reported roles. First, apart from the traditional function in motor control, the SMA plays a critical role in processing of speech communication and language reception; for example, the use of inner speech mechanisms during language encoding, lexical disambiguation, syntax and prosody integration, and context-tracking [77]. In addition, a meta-analytic review [82] has shown that left putamen co-activates with the brain regions directly involved in language processing. Therefore, the CA functional connectivity related to pragmatic language deficits might be attributed to its functions in the memory-language interface and social cognition as well as its integration with the SMA and putamen, which are involved in language processing.

When it comes to rigid personality paralleling the restricted and repetitive domain of autism, our results extend previous studies by showing that increased connectivity between the right CeA and the left inferior lateral occipital cortex was associated with rigid personality, proposing that central amygdala-visual integration might be one potential neural circuit underlying the repetitive behaviors. Specifically, autistic individuals have been reported to exhibit increased functional connectivity between the CMA and the occipital cortex compared to typically-developing individuals [55]. One recent study [50] has shown that increased CeA volume is associated with more severe repetitive behaviors. It has been shown that the CeA participates in visual stimulus processing [83], and that atypical recruitment and regulation of the visual cortex as well as atypical visual attention are related to restricted and repetitive behaviors [84]. Nevertheless, further investigation is needed to improve our understanding of this neural mechanism associated with the pathogenesis of repetitive behaviors.

In contrast to our hypothesis drawing on a previous finding [50] that increased CeA volume was linked to rigid personality, we found that decreased left hippocampal CA1 volume was associated with rigid personality. This discrepancy may be due to the aforementioned limitations of the previous study [50], but an alternative explanation is that the neural correlates of rigid personality do not fully overlap with those of restricted and repetitive behaviors; restricted and repetitive behaviors comprise different subtypes (including restricted, stereotyped, ritualistic, sameness and self-injurious) [85], whereas rigid personality relates more specifically to a desire for sameness [7]. In view of hippocampal CA function, it is evident that hippocampal CA1 neurons play an important role in the retrieval of episodic memory and reversal learning [86, 87]. A high-resolution MRI study [37] also showed that decreased hippocampal CA1 volume was associated with retrieval deficits.

Apart from volumetric alterations, we found that increased connectivity between the left hippocampal CA3 and the right superior parietal lobule was associated with rigid personality; the involvement of the parietal lobe might be explained by hyper-reactivity to sensory inputs seen in restricted and repetitive behaviors [1]. Another possible explanation is functional interplay between the hippocampus and the parietal lobe; for example, the hippocampal CA3 plays a role in the retrieval of episodic contexts and the parietal lobe also activates during episodic memory retrieval [88, 89]. In addition, we found that increased connectivity between the right hippocampal CA4 and the left superior lateral occipital cortex and increased connectivity between the right hippocampal DG and the left superior lateral occipital cortex were associated with rigid personality. Hippocampal DG volume has been associated with visual-spatial memory retrieval [90]. Our result suggests that hippocampal CA4 and DG might share a common neural circuit underlying rigid personality, possibly due to the lack of a clear distinction between the DG and CA4 in terms of their cytoarchitecture [91]. For example, certain anatomists divided the DG into fascia dentata and CA4 [92], and cytoarchitectonic mapping of the hippocampus in a human post-mortem brain also identified the hippocampal formation as composed of CA1, CA2, CA3, and DG (including fascia dentata and CA4) [23].

Our results about the correlations of rigid personality with decreased CA1 volume and increased functional connectivity of the CA3, CA4, DG appear to challenge the traditional view that the hippocampus contributes exclusively to memory and learning. Rigidity, defined as difficulty adjusting to new situations or altered routines, reflects a desire for sameness and the presence of inflexibility and over-conscientiousness [7, 93, 94]. Although it is an unexpected finding, the involvement of the hippocampal subfields in rigid personality might be explained by the links between rigid patterns of behavior observed in autistic individuals and their atypical retrieval and learning [35, 36, 95]. Specifically, it has been proposed that distortion is a characteristic of human memory and that some memory errors can be adaptive for flexible behaviors; conversely, overly rigid, robust, and undistorted memory may restrict the modification of thinking and result in low relational binding and behavioral inflexibility [96]. There is evidence from human research that less flexible and less relational retrieval and learning may sometimes cause restricted and repetitive behavior, or, conversely, that inflexible behaviors in autistic individuals may result in rigid and less connected memories [36]. Therefore, the present finding provides a clue that hippocampal CA1, CA3, CA4 and DG are implicated in rigid memory intertwining rigid behavior, suggesting a promising research direction for future studies to advance our understanding of their distinct roles.

Finally, across the results, it was somewhat surprising that a single brain region exhibited associations with different subdomains of BAP depending on its structure and function. For example, CA1 volume, but not its functional connectivity, was associated with rigid personality, whereas CA1 functional connectivity, but not its volume, was associated with pragmatic language deficits. This finding indicates that hippocampal resting-state functional connectivity patterns perhaps capture the current state of social language usage better than hippocampal volume. It is possible that hippocampal contribution to pragmatic language requires the functional activation of other brain regions that are involved in social cognition, whereas the role of the hippocampus in rigid personality may be less dependent on its coactivation with other structures and more narrowly related to hippocampal involvement in atypical memory and learning. Such divergence of the results also emphasizes the necessity to understand the relationship between brain structure and function. One view in favor of brain structure and function being related suggests that the structural properties (e.g., volume, thickness, surface area and curvature) of a region, as well as the white matter tracts, can influence the patterns and strength of its functional activation [97]. Notably, functional connectivity is also observed between regions in the absence of structural connectivity [98]. Evidence also demonstrates that structure and function display unique features (e.g., associations with functional activity but no associations with volume or white matter tract integrity) that vary with cognition and behavior [99], in support of our results of divergent CA1 structural and functional patterns in the context of rigid personality and pragmatic language deficits.

There are several limitations that should be considered. One methodological limitation is the length of the functional MRI scan, which was 5 minutes in our protocol. Since it has been shown that the reliability of resting-state functional connectivity can increase for durations longer than 6 minutes [100], this scan duration was not ideal. The sample is also small due to loss to follow-up during the 19-year period since the birth of the participants. In addition, the socioeconomic status of the families in this study is higher than the median of the population in the region from which they were recruited in 1998, such that the current results may not extend to PNMS-exposed individuals from lower socioeconomic backgrounds. Further, like the prevalence of autism (e.g., 4 males for every 1 female diagnosed) [101], BAP traits tend to aggregate more often in male relatives than female relatives [102–106], however, 65.6% of our sample were females, which may limit comparability with autism studies that included predominantly male participants. Another limitation is the potential confounding effects of PNMS on the observed relationships between BAP traits and amygdala and hippocampal volumes and functional connectivity. To address this, we applied a residualization approach to isolate the PNMS-related variance. After residualization, the association with right CMA volume became non-significant, whereas the associations with hippocampal volume as well as amygdala and hippocampal functional connectivity remained significant, supporting the robustness of our findings. A further limitation is the absence of a control group with similar levels of BAP traits but without a prenatal stressor. Without such a comparison, it remains unclear whether the neural markers identified in this unique context can generalize to typical autism populations, potentially limiting their diagnostic or therapeutic relevance. Therefore, the results need to be replicated in non-PNMS-exposed autistic samples.

Despite the limitations, some strengths are inherent in this study. One strength is that BAP, a form of “broader” autism, was examined within a non-clinical sample that, possibly as a result of prenatal exposure to a natural disaster, was found to have a wide range of autistic-like traits [16]. Use of this sample, which endured a range of severity of prenatal stress, extends our conventional understanding of BAP from previous studies of, for example, first-degree relatives of children with autism [7, 56]. A second strength of this study is our examination of the three core broad autism phenotypes separately: aloof personality, pragmatic language deficits and rigid personality [10]. A third strength is the investigation of specific amygdala nuclei (i.e., BLA and CMA) and hippocampal subfields (i.e., CA1, CA3, CA4 and DG), recognizing that the amygdala and hippocampus are composed of heterogenous structures that may be differentially associated with specific broad autism phenotypes. Although the BAP assessment and the functional MRI scanning occurred concurrently at age 19, the neural correlates observed in this non-clinical sample are potential biomarker candidates that require further development in future research. Further, since the children at ages 16 and 19 self-reported their BAP phenotypes and underwent structural and diffusion MRI scanning, this design enables us to examine longitudinal changes in relationships between three BAP subdomains and amygdala and hippocampal volume and structural connectivity from age 16 to age 19.

In conclusion, our results suggest that hippocampus-motor connectivity, which is responsible for emotion, memory and motor processing, possibly contributes to pragmatic language as seen in young adults. As well, our study suggests that hippocampal volume and amygdala-occipital and hippocampus-occipital-parietal connectivity, which are responsible for emotion, memory, visual and sensory processing, possibly contribute to rigid personality in young adulthood. Nonetheless, the interpretation of these findings warrants caution, as they are derived from a stress-exposed population and lack a control group. In addition, in the context of previous findings on the effects of PNMS on autistic-like traits [16, 17], future analyses will test any mediating role of brain structure and function in explaining any possible neural mechanisms by which PNMS predicts autistic-like traits in human offspring.

## Supplementary information

Table S1 and Figs. S2-S5

## Data Availability

The data supporting the findings of this study are available from the corresponding author upon reasonable request.
