# Supplementary material for: Amygdala and hippocampal contributions to broad autism phenotype: Project Ice Storm
Source: Transl Psychiatry. 2026 Mar 19;16:184. doi: 10.1038/s41398-026-03918-6 (PMC13039455; doi:10.1038/s41398-026-03918-6)

Table S1. Associations between brain measures and BAP scores before and after controlling for prenatal maternal stress.

| BAP traits | Brain | Original model | Residualized model (Residualized for PNMS) |  |
| --- | --- | --- | --- | --- |
| Pragmatic language | Volume |  |  |  |
|  | Right CMA volume | Beta = 0.365, B = 7.533, se = 3.536, p = 0.042 | Beta = 0.280, B = 6.195, se = 3.930, p = 0.126 |  |
|  | Right MeA volume | Beta = 0.385, B = 3.676, se = 1.631, p = 0.032 | Beta = 0.345, B = 3.524, se = 1.773, **p = 0.056** |  |
| Pragmatic language | Functional connectivity |  |  |  |
|  | | Left CA1-left SMA | Beta = -0.715, B = -0.126, se = 0.023, p < 0.001 | Beta = -0.716, B = -0.122, se = 0.022, **p < 0.001** |
|  | | Right CA4-left putamen | Beta = 0.735, B = 0.126, se = 0.020, p < 0.001 | Beta = 0.689, B = 0.123, se = 0.021, **p < 0.001** |
| Rigid personality | | Volume |  |  |
|  | | Left CA1 volume | Beta = -0.406, B = -37.843, se = 15.755, p = 0.023 | Beta = -0.478, B = -48.160, se = 16.552, **p = 0.007** |
| Rigid personality | | Functional connectivity |  |  |
|  | | Right CMA-left iLOC | Beta = 0.708, B = 0.109, se = 0.019, p < 0.001 | Beta = 0.666, B = 0.107, se = 0.021, **p < 0.001** |
|  | | Right CeA-left iLOC | Beta = 0.705, B = 0.106, se = 0.019, p < 0.001 | Beta = 0.657, B = 0.103, se = 0.021, **p < 0.001** |
|  | | Right CA4-left sLOC | Beta = 0.725, B = 0.131, se = 0.024, p < 0.001 | Beta = 0.709, B = 0.140, se = 0.026, **p < 0.001** |
|  | | Right DG-left sLOC | Beta = 0.734, B = 0.124, se = 0.022, p < 0.001 | Beta = 0.725, B = 0.135, se = 0.024, **p < 0.001** |
|  | | Left CA3-right SPL | Beta = 0.724, B = 0.109, se = 0.019, p < 0.001 | Beta = 0.662, B = 0.103, se = 0.021, **p < 0.001** |

*BAP* broad autism phenotype*, CA* cornu ammonis, *CeA* central nucleus of the amygdala, *CMA* centromedial nuclei of the amygdala, *CA* cornu ammonis, *DG* dentate gyrus, *iLOC* inferior lateral occipital cortex, *MeA* medial nucleus of the amygdala, *PNMS* prenatal maternal stress, *SMA* supplementary motor area, *sLOC* superior lateral occipital cortex, *SPL* superior parietal lobule

Fig. S2. Correlations of amygdala and hippocampal subregion volumes with two broad autism phenotypes independent of prenatal maternal stress.


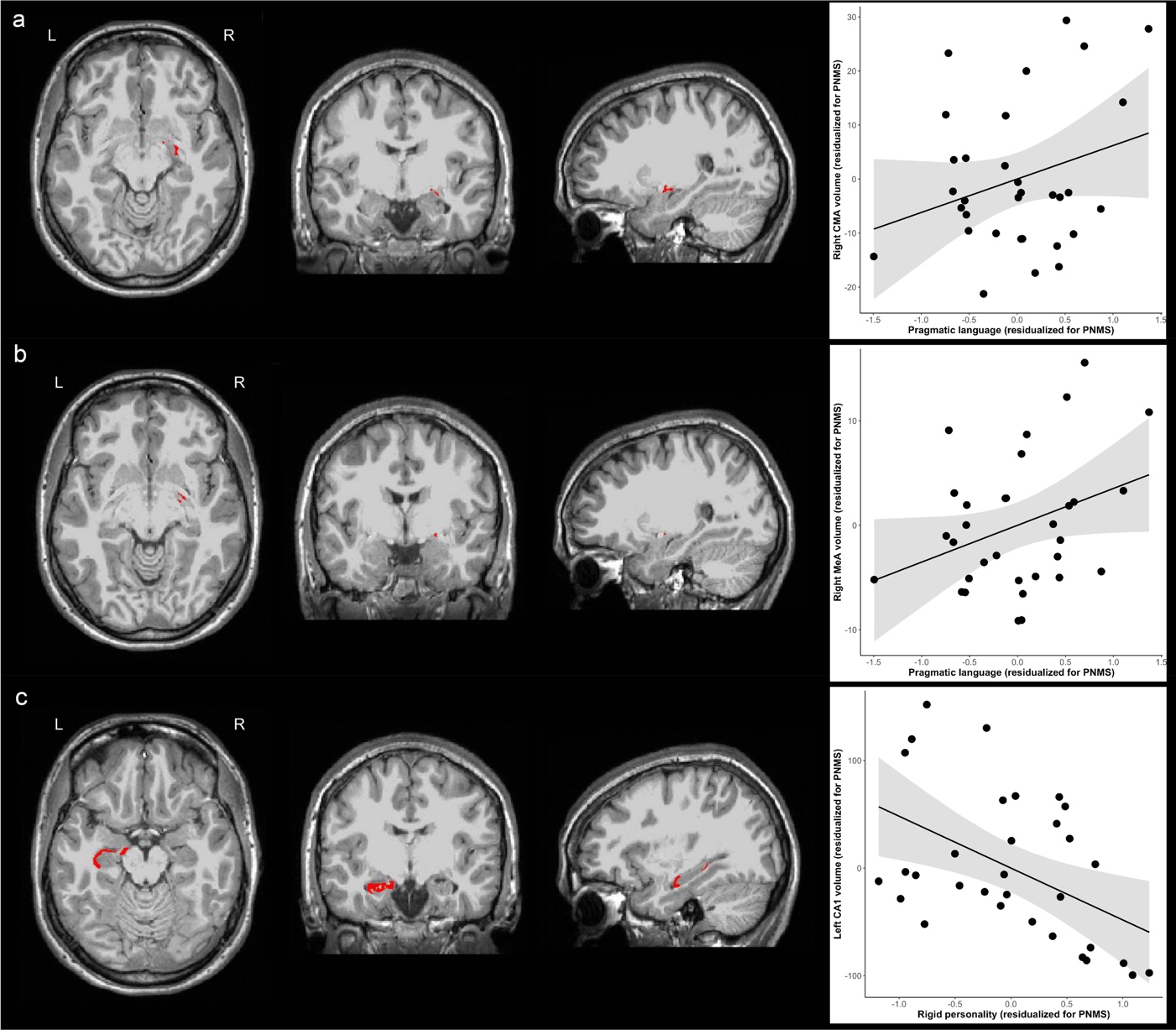


Fig. S3. Correlations of amygdala subregion functional connectivity with rigid personality independent of prenatal maternal stress.
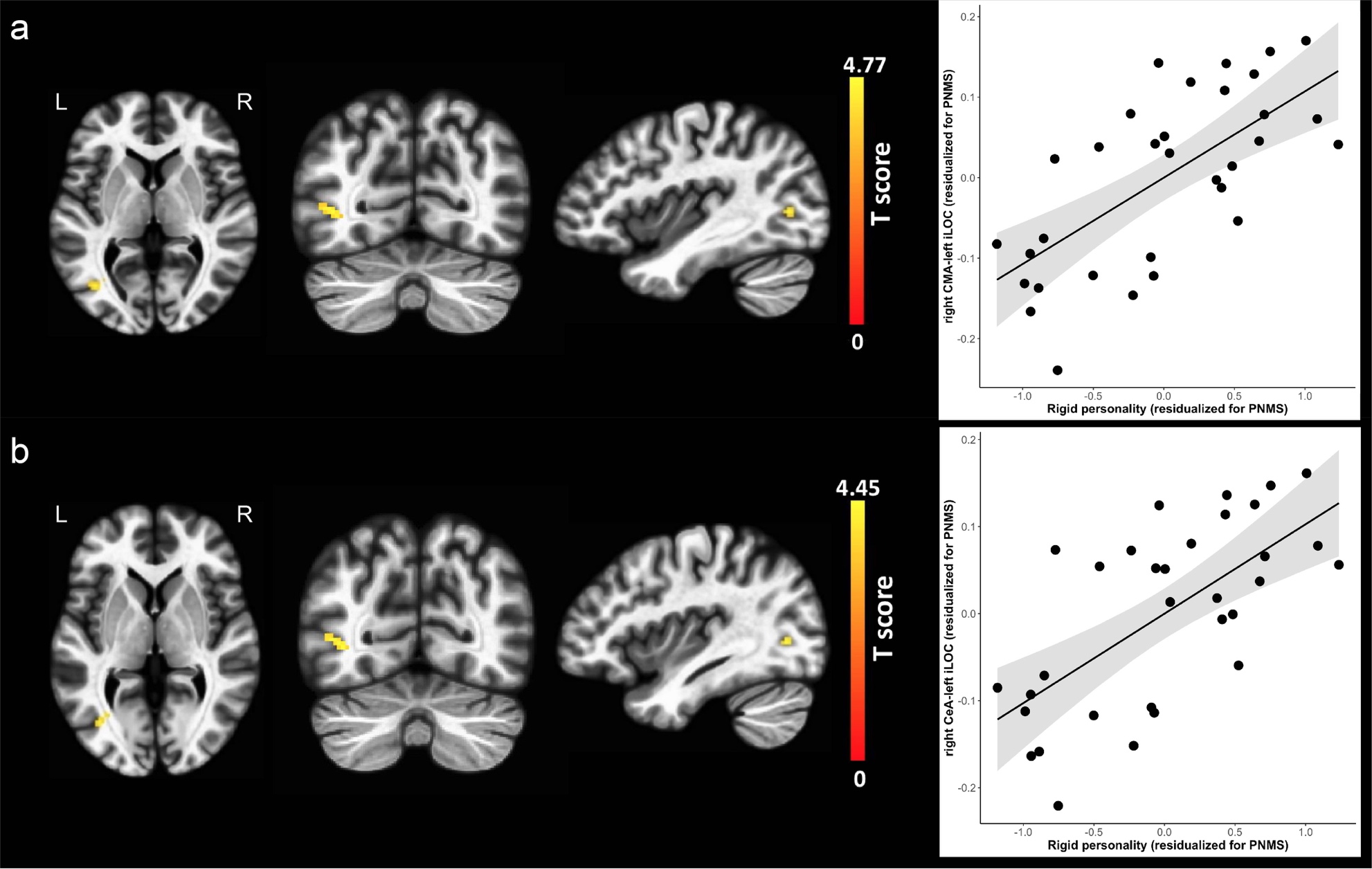


Fig. S4. Correlations of hippocampal subregion functional connectivity with pragmatic language impairment independent of prenatal maternal stress.


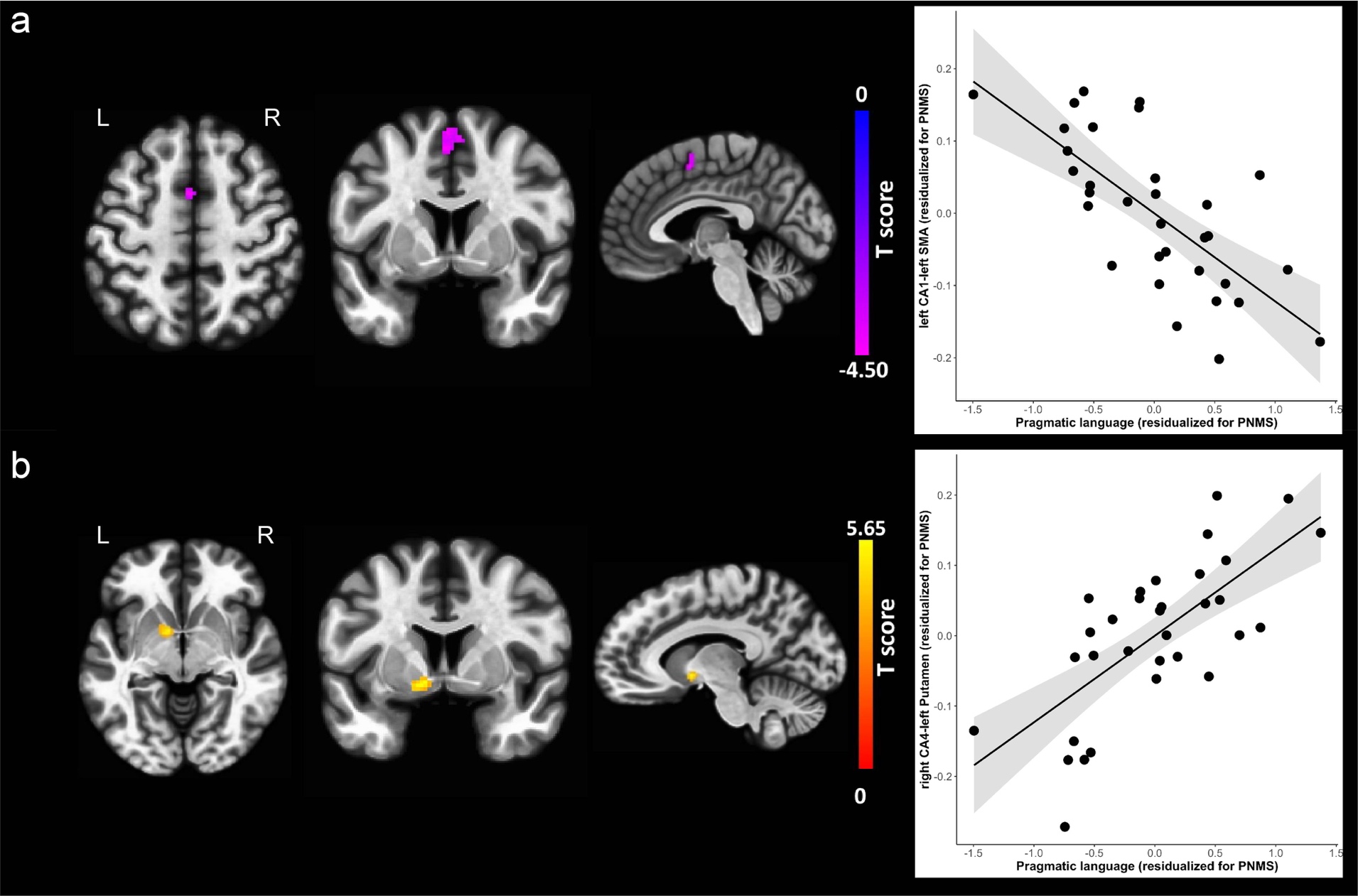


Fig. S5. Correlations of hippocampal subregion functional connectivity with rigid personality independent of prenatal maternal stress.


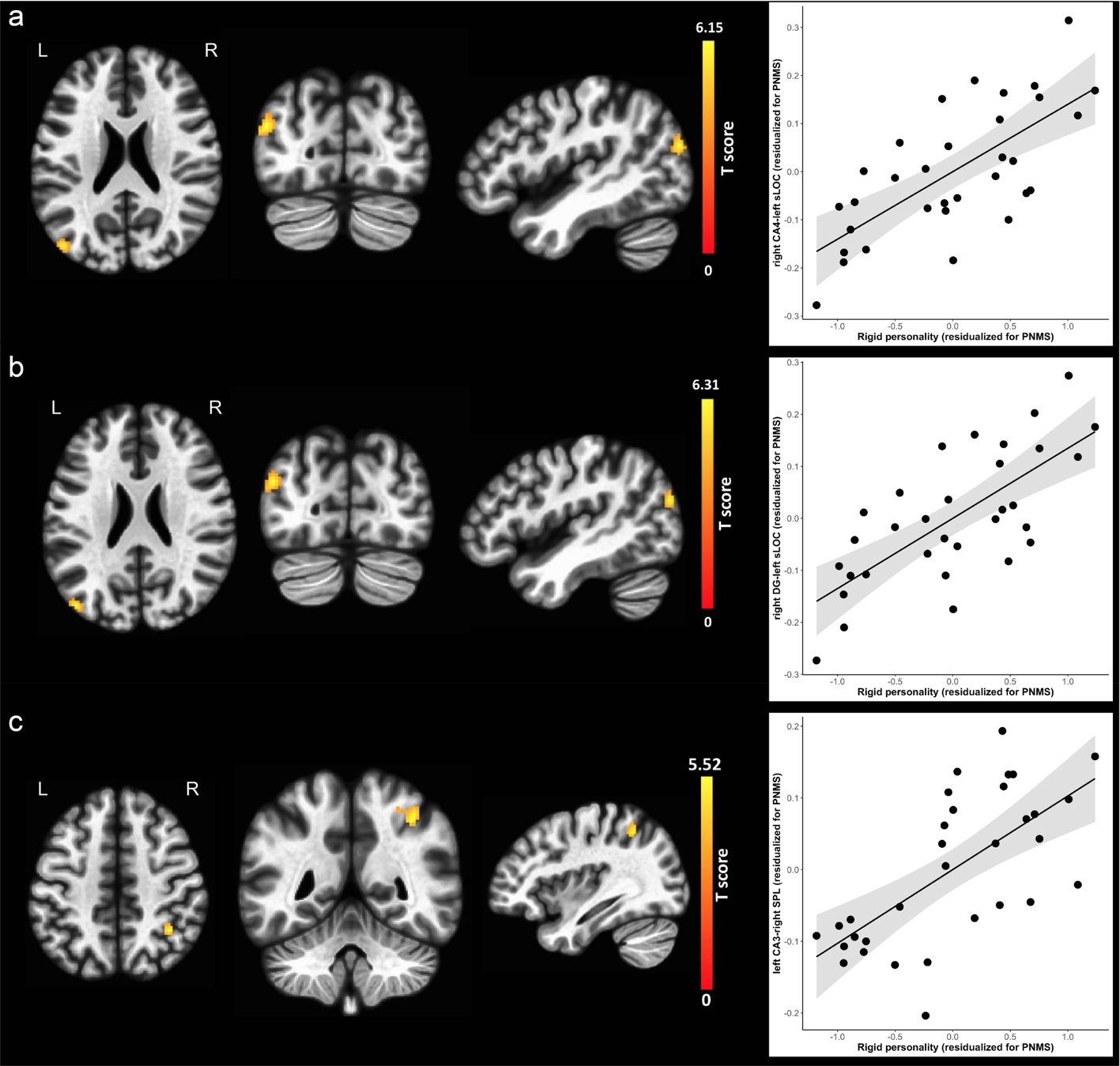

Supplement: Supplementary file 1 — Table S1 and Figs. S2-S5 [file 41398_2026_3918_MOESM1_ESM.docx]
